# Elastic Polyurethane as Stress‐Redistribution‐Adhesive‐Layer (SRAL) for Directly Integrated High‐Energy‐Density Flexible Batteries

**DOI:** 10.1002/advs.202401635

**Published:** 2024-06-03

**Authors:** Yige Xiong, Zhongjie Wang, Xiaohui Yan, Taibai Li, Siqi Jing, Tao Hu, Huixin Jin, Xuncheng Liu, Weibo Kong, Yonglin Huo, Xiang Ge

**Affiliations:** ^1^ Department of Materials and Metallurgy Guizhou University Guiyang Guizhou 550025 P. R. China; ^2^ College of Polymer Science and Engineering Sichuan University Chengdu 610065 P. R. China

**Keywords:** direct integration, finite element analysis, flexible, Li‐ion batteries, stress redistribution

## Abstract

The low mechanical reliability and integration failure are key challenges hindering the commercialization of geometrically flexible batteries. This work proposes that the failure of directly integrating flexible batteries using traditional rigid adhesives is primarily due to the mismatch between the generated stress at the adhesive/substrate interface, and the maximum allowable stress. Accordingly, a stress redistribution adhesive layer (SRAL) strategy is conceived by using elastic adhesive to redistribute the generated stress. The function mechanism of the SRAL strategy is confirmed by theoretical finite element analysis. Experimentally, a polyurethane (PU) type elastic adhesive (with maximum strain of 1425%) is synthesized and used as the SRAL to integrate rigid cells on different flexible substrates to fabricate directly integrated flexible battery with robust output under various harsh environments, such as stretching, twisting, and even bending in water. The SRAL strategy is expected to be generally applicable in various flexible devices that involve the integration of rigid components onto flexible substrates.

## Graphical abstract

Simultaneously realizing high electrochemical performance and mechanical reliability are crucial for flexible batteries. Herein, the authors propose a stress redistribution adhesive layer (SRAL) strategy that can align the distribution of generated stress and allowable stress, thus providing a general strategy to enhance mechanical reliability for directly integrated flexible battery.

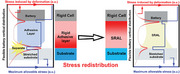

## 1 Introduction

With the continuous development of flexible electronic devices such as electronic skin,^[^ ^1^ ^]^ flexible displays,^[^ ^2^ ^]^ and wearable medical devices,^[^ ^3^ ^]^ higher demands are being placed on their energy storage systems.^[^ ^4^ ^]^ The next generation of energy storage devices is required to meet not only a variety of demands on performances, such as high energy density, and high reliability, but also stringent usage scenarios including bending, violent shaking and exposure to moisture.^[^ ^5^ ^]^ Among the existing energy storage devices, lithium‐ion batteries are considered the primary candidate due to their high energy density, well‐established industrial production protocols and outstanding performance, while the development of flexible lithium‐ion batteries is more challenging than other flexible components because of their vulnerability to moisture and complex configuration.^[^ ^6^ ^]^

Extensive research has been conducted on realizing the flexibility of lithium‐ion batteries, with the most common strategies being intrinsic flexible batteries prepared using intrinsic flexible materials or geometric flexible batteries enabled by macroscopic structures.^[^ ^7^ ^]^ Intrinsic flexible batteries require each component to be an intrinsically flexible material, such as carbon nanotube current collectors,^[^ ^8^ ^]^ soft gel electrolytes,^[^ ^9^ ^]^ and intrinsically flexible electrodes.^[^ ^10^ ^]^ This enables intrinsic flexible batteries to exhibit omnidirectional flexibility. However, intrinsic flexible batteries inevitably incorporate a significant amount of electrochemically non‐active components, resulting in a significant decrease in energy density.^[^ ^7^, ^11^ ^]^ Additionally, due to the extreme sensitivity of lithium‐ion batteries to moisture and oxygen, intrinsically flexible package materials introduce challenges for long‐term stability. In contrast, geometrically flexible batteries adopt a macrostructure, with rigid energy storage units integrated on a flexible substrate to achieve the overall flexibility of the flexible battery. The manufacturing of rigid energy storage units is compatible with the current packaging protocol, thus allowing effective sealing. Consequently, geometric flexible batteries are considered more promising for practical applications.^[^ ^7a^ ^]^

In 2013, a geometrically flexible (island‐bridge structured) lithium‐ion battery was reported.^[^ ^12^ ^]^ The rigid energy storage units composed of conventional electrodes could provide higher electrochemical performance compared to intrinsically flexible batteries. Thereafter, various strategies (patterned structure,^[^ ^13^ ^]^ biomimetic structure,^[^ ^14^ ^]^ mechano‐graded,^[^ ^11a^ ^]^ etc.) have been developed to improve the electrochemical or mechanical performance of such geometrically flexible batteries.^[^ ^14^, ^15^ ^]^ Still, for lithium‐ion batteries which are sensitive to moisture, it is challenging to manufacture flexible batteries with comparable environmental stability to commercialized rigid batteries due to the lack of reliable packaging strategies. Directly integrated flexible batteries, which are manufactured by directly integrating commercialized coin cells on flexible substrates, are expected to provide highly reliable electrochemical performance because the metallic packaging shell shows much better air impermeability than soft packaging materials.^[^ ^16^ ^]^ However, the direct integration process based on conventional rigid epoxy‐type glue cannot provide satisfactory robustness because the stress generated during deformation would concentrate at the glue/substrate interface, which can only tolerate small stress because the surface of common flexible substrates (for example, PDMS, Ecoflex or SEBS) are usually inert and cannot form strong binding with most glues (Figure S1, Supporting Information). The further understanding and optimization of the mechanical behavior of directly integrated flexible batteries are of high significance in meeting the demand for practical flexible electronics.

Herein, we propose the robust integration of commercial coin cells onto flexible substrates into a flexible battery system can be realized by using elastic adhesive, which functions as the stress‐redistribution‐adhesive‐layer (SRAL). Based on the idea of geometrically flexible battery preparation, the flexible battery system prepared using the SRAL strategy can maintain the high energy density of commercial batteries while achieving geometrical flexibility, and achieve the direct integration of high energy density flexible batteries. With fundamental analysis using the Finite Element Analysis (FEA) method, we demonstrate that the SRAL enables direct, and reliable integration of rigid energy storage components with flexible substrates by redistributing the maximum stress from the weaker adhesive/substrate to the stronger cell/adhesive contact interface. As a proof‐of‐concept, we synthesized a polyurethane (PU) by polymerizing IPDI, PTMG, and BDO, resulting in the production of a flexible adhesive (Max stretch of 1425%, Young's Modulus is ≈0.1217 MPa). The abundant NHCOO‐ groups and the cross‐linked entanglement between PDMS and PU contribute to strong adhesion at the interface of cell/adhesive and adhesive/flexible substrate. Besides, A flexible battery made of arrays of LR41 coin cells integrated onto various types of flexible substrates (PDMS, Ecoflex and SEBS) using SRAL is demonstrated to show comparable mechanical properties to those batteries integrated using conventional rigid adhesive under vertical stretching (maximum allowable stress for SRAL vs Epoxy:10.6 vs ≈8.5 KPa on PDMS, 301.0 vs ≈18.3 KPa on Ecoflex, and 522.2 vs ≈286.4 KPa on SEBS, respectively), but improved robustness under various complex environments including bending, violent shaking or immersing in deionized water by redistributing the maximum generated stress out of the weak adhesive/substrate interface. This work is expected to provide theoretical guidance and a general strategy for aligning the maximum produced stress and the maximum allowable stress in the device, thus pushing the design, and fabrication of directly integrated flexible batteries for high‐end flexible electronics.

## 2 Results

### 2.1 Design Principles of Directly Integrated Flexible Battery Based on SRAL

**Figure 1:**
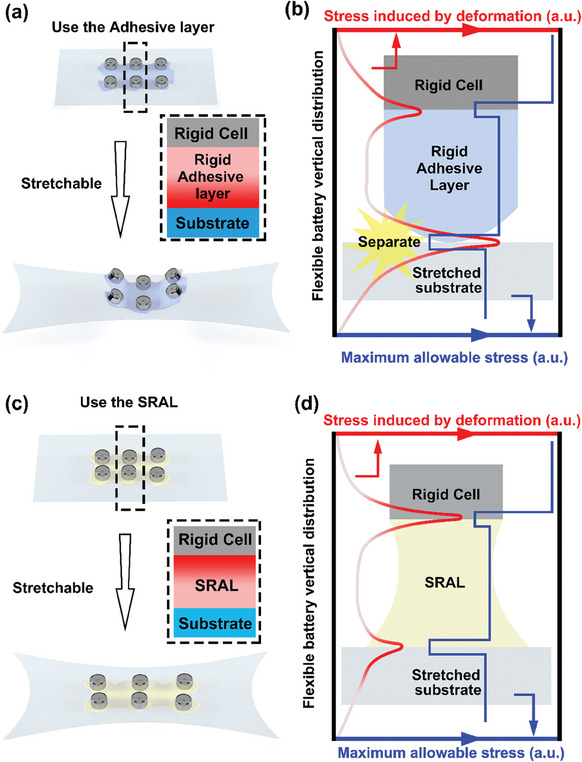
Design Principles of SRAL. a,b) The flexible battery integrated using conventional rigid adhesive layer would concentrate the stress at the weak substrate/adhesive layer interface, leading to unmatched distribution between the allowable stress and produced stress. c,d) For flexible batteries prepared using stress‐redistribution‐adhesive‐layer (SRAL), the maximum stress is redistributed to the relatively more robust interface between the stainless steel coin cell and the adhesive layer.

For geometrically flexible batteries manufactured by directly integrating commercialized coin cells onto flexible substrates, mechanical reliability is the key for the device to work under complex deformation scenarios.^[^ ^16^ ^]^ Various patterned structures including spine‐like, kirigami‐inspired, and accordion‐like have been proposed to alleviate the stress in the inter‐cell spaces, while the adhesion at the cell/substrate using conventional glue is still vulnerable.^[^ ^6^, ^14^, ^17^ ^]^ Fundamentally, as shown in **Figure** **1**, the adhesion of coin cells onto flexible substrates using adhesives generates two interfaces (cell/adhesive and adhesive/substrate). When using conventional rigid glues (taking epoxy as an example) as the adhesive (Figure 1a), the generated stress during deformation would maximize at the adhesive/substrate interface (Figure 1b). This interface is usually weak because most flexible substrates expose inert surfaces and cannot form strong binding with the adhesive layer. Consequently, a mismatch between the generated stress and the maximum allowable stress would occur, thus leading to subsequent failure at the adhesive/substrate interface. Intuitively, the mechanical performance of the device can be improved if the distribution of the generated stress can align with the strength of the device with proper mechanical properties on each part (Figure 1c). Instead of using rigid glue, we conceive that an elastic adhesive could perform as the SRAL, with which the maximum stress generated during deformation would be transferred out of the weak adhesive/substrate interface (Figure 1d), thus improving the overall mechanical reliability for directly integrated flexible batteries.

**Figure 2:**
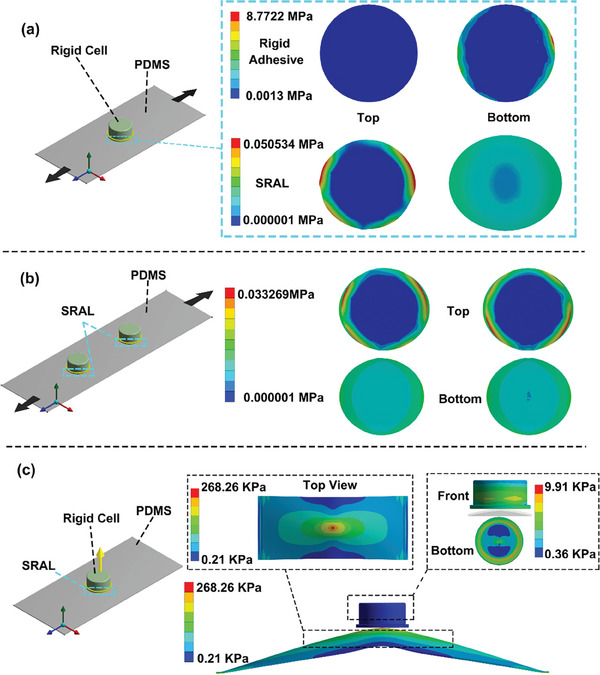
Finite element analysis of SRAL. a) The stress distribution at the top and bottom of the adhesive layer. (a) emphasizes the stress maximizes at the bottom (substrate/adhesive layer interface) when using conventional rigid adhesive while the stress redistributes and maximizes at the top (rigid cell/adhesive layer interface) when using SRAL. b) Illustrates the stress distribution of a flexible battery array prepared using SRAL under lateral deformation. c) Demonstrates the stress distribution of both the SRAL and the flexible substrate during vertical deformation.

To quantify the effectiveness of the SRAL strategy, we conducted the finite element analysis (FEA) to compare the stress distribution when using rigid adhesive or elastic SRAL during integration. The Young's modulus and Poisson's ratio values of rigid adhesive material were set as 1000 MPa and 0.39, respectively. For PDMS, Young's modulus of 2.6692 MPa and Poisson's ratio of 0.499 were used. For SRAL, the Young's modulus was set as 0.12 MPa, and the Poisson's ratio was set as 0.48. For simulating the tensile deformation, the left side of the substrate was fixed while a displacement of 10 mm was applied to the right side. The imprint method was employed to record the stress distribution on the contact surface including both the cell/adhesive and adhesive/substrate interfaces. We first compared the stress distribution at the two interfaces when using rigid glue or SRAL as the adhesive (**Figure** **2**a) when stretching a flexible substrate integrated with a single cell. For rigid glue, the stress on the upper contact surface (cell/rigid adhesive) was negligible (≈0.0013 MPa). However, the stress distribution on the lower contact surface (rigid adhesive/substrate) was non‐uniform and maximized at the edges (8.7722 MPa). In contrast, when using the SRAL for integration, the stress is distributed at the upper contact surface (cell/SRAL), and maximized at the edges (0.0505 MPa). In this case, the stress on the lower contact surface (SRAL/substrate) was negligible (Figure 2a). We further examined the stress distribution when the flexible device was integrated with multiple cells (Figure 2b). The stress distribution at each interface corresponded well to the scenario when the device was integrated with a single cell, confirming the general effectiveness of the SRAL strategy. We further employed the cohesive zone method (CZM) to simulate the crack propagation process of a rigid battery undergoing upward displacement until detachment. First, we defined two types of CZM materials. For the upper contact interface (cell/adhesive) with stronger adhesion, we set a maximum normal contact stress of 1 MPa, and a critical fracture energy for a normal separation of 1 Jm^−2^. For the lower contact interface (adhesive/substrate) with weaker adhesion, the maximum normal contact stress was set to 5 KPa, and the critical fracture energy for normal separation was set to 5 Jm^−2^. For both CZMs, the artificial damping coefficient of 1 ms was set for mathematical stability during simulation. Under the same condition, the flexible battery integrated using SRAL or the rigid adhesive layer showed a similar failure mechanism (cracks initiated from the edge of the adhesive/substrate interface and propagated inward) (Figure 2c; Figure S2, Supporting Information). Therefore, redistributing the maximum generated stress out of the weaker adhesive/substrate interface during deformation should be effective for improving the robustness of such a directly integrated flexible battery when the device is deformed in other directions.

### 2.2 General Effectiveness When Using PU as the SRAL on Various Flexible Substrates

**Figure 3:**
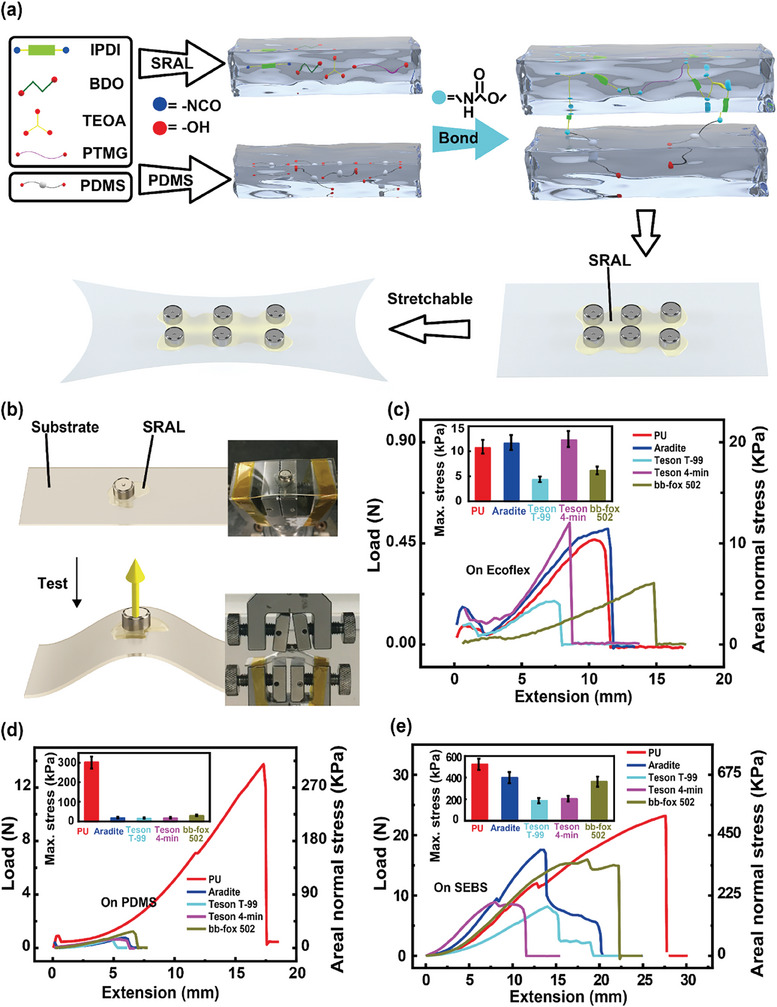
The preparation process of PU‐SRAL and the vertical deformation test. a) Schematic illustration showing the synthesis of elastic PU as the SRAL for integrating arrays of coin cells onto flexible substrate. b) The strain/stress can be quantified by vertical deformation on a mechanical tester. c–e) Vertical deformation test results of flexible batteries prepared using different substrates and different adhesive layers.

To verify the SRAL strategy, polyurethane (PU) was synthesized as the elastic adhesive. PU was a tunable block copolymer that could achieve different properties by adjusting the ratio of soft and hard segments.^[^ ^18^ ^]^ In this work, isophorone diisocyanate (IPDI) and BDO were selected as the hard segment in the polyurethane chain. It provided a large number of NCO groups to react with OH to form NHCOO‐ groups, which provided strong adhesion to various flexible rubbers, and metallic cells. The long‐chain macromolecule PTMG used provides excellent mechanical flexibility for polyurethane materials. **Figure** **3**a briefly illustrates the synthesis of polyurethane‐based SRAL. First, IPDI, poly(tetramethylene glycol) (PTMG), and 1,4‐butanediol (BDO) were reacted for chain extension.^[^ ^19^ ^]^ Then, triethanolamine (TEOA) was added. Before solidification, the semi‐polymerized polyurethane was dripped onto the desired regions of a semi‐cured flexible substrate. The cells were placed on the semi‐polymerized PU layer and gently tapped to remove excess bubbles. The system was then kept at 80 °C for 24 h for the PU to cure. As the curing reaction progresses, a cross‐linked molecular entanglement forms between PDMS and PU, thus significantly enhancing the adhesion between PDMS and PU. Meanwhile, FTIR indicates that no new chemical bonds are generated between PDMS and PU (Figure S3, Supporting Information). The structural composition, thermodynamic properties and mechanical properties of PU were characterized by FTIR (Figure S3, Supporting Information), TG‐DTG (Figure S4, Supporting Information) and tensile test (Figure S5, Supporting Information), and the results showed that PU remained stable below 275 °C and had excellent mechanical properties, Young's modulus was ≈0.1217 MPa.

To quantify the adhesion for the adhesive/substrate interface for such a directly integrated flexible battery (Figure 3b), its flexible substrate was fixed at the bottom of a tensile testing machine, while the cell (integrated onto the flexible substrate) was clamped by the tensile mold. The specimen was then stretched upward until detachment. For systematic comparison, three common flexible substrates including PDMS, Ecoflex, and SEBS were coupled with our PU as well as other four commercial adhesives including Aradite, Teson T‐99, Teson 4‐min, and bb‐fox 502 (Figure 3c–e). It's worth noting that all tested devices fail at the lower adhesive/substrate interface, confirming that redistributing the maximum stress out of this interface was useful to improve the robustness of the device under other deformation modes (lateral stretching, for example). Compared with other commercial adhesives, the adhesion of PU on various substrates showed first‐tier performance and was particularly better than epoxy on PDMS (301.0 KPa vs 20–30 KPa). Additionally, the maximum allowable stress for PU (or epoxy) on PDMS was similar to the results calculated using finite element analysis software (301.0 vs 268.26 KPa), confirming the reliability of the CZM data used for simulation. These results demonstrate that the elastic PU binder could provide comparable or better adhesion than commercialized adhesives at the adhesive/substrate interface, while the elastic property of PU could redistribute the maximum generated stress out of this adhesive/substrate interface, thus functioning as SRAL for improving the overall mechanical reliability of the directly integrated flexible battery.

### 2.3 Electrochemical Demonstration of Flexible Batteries Based on SRAL

**Figure 4:**
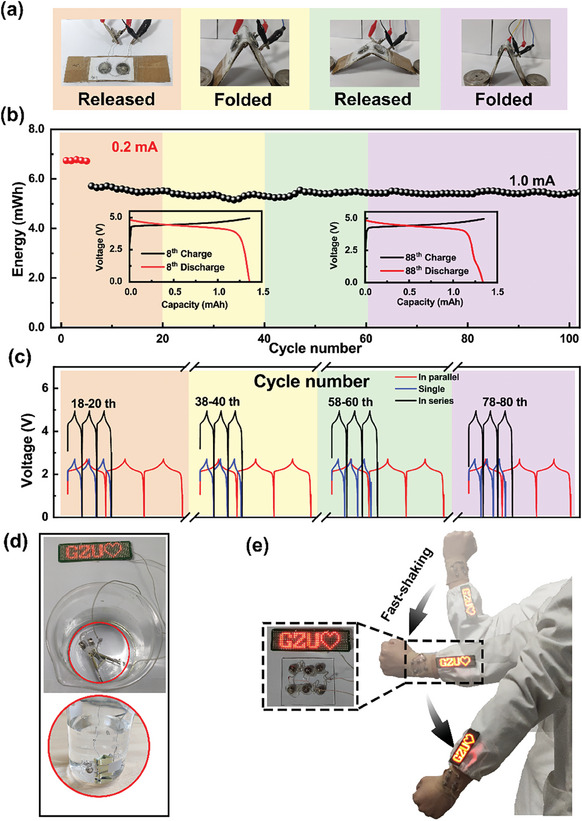
Robust electrochemical performance of the flexible batteries integrated using SRAL. a) Schematic diagram of the deformation of a flexible battery integrated using SRAL (released/folded) b) Flexible batteries using SRAL showed high stability under cycling at 1.0 mA current (whether bent or not). c) The stability of series and parallel flexible battery integrated using SRAL. The charge/discharge curves show the device deliver robust output with or without bending. d,e) The flexible battery integrated using SRAL delivers stable output under complex conditions (bending in water, violent shaking).

The stable output under complex working conditions is the key for the flexible battery to meet the demand for various flexible electronics.^[^ ^20^ ^]^ To evaluate the electrochemical performance of the directly integrated flexible battery using the SRAL strategy, we directly integrated multiple CR2032‐type NCM/LTO cells (average loading is 13.0 ± 2.0 mg NCM vs 12.0 ± 2.0 mg LTO) in series or parallel onto PDMS substrates (6.0 × 2.0 × 0.1 cm^3^) using the prepared PU (**Figure** **4**a,b). The structures (Figures S6 and S7, Supporting Information) and half‐cell electrochemical performance (Figures S8–S11, Supporting Information) of the NCM and LTO raw materials were tested. The devices are stabilized at 0.2 mA for five cycles and then cycled at 1.0 mA under repeated bending/releasing. For each NCM/LTO full cell, the loading of NCM is 12.0 ± 2.0 mg and 10% excessive anode was used. The similar charge/discharge curves at the 8th and the 88th cycle (inset in Figure 4b) indicate the robust electrochemical performance of the device upon bending. To further confirm the good electrical contact during deformation, we compared the charge–discharge curves of single cells, two cells in series as well as two cells in parallel during cycling with repeated deformation (Figure 4c). The battery in series shows doubled output voltage and the battery in parallel shows doubled capacity compared to the single cell, indicating the reliable connection within our directly integrated flexible battery. To visualize the robustness of the flexible battery integrated using SRAL, we have used smaller LR41‐cells as units to be integrated on PDMS (6.0 × 5.0 × 0.1 cm^3^) to form a directly integrated flexible battery to demonstrate the viability of fabricating directly integrated batteries with higher density of integration level (Figure 4d). Compared with the use of rigid adhesives, this directly integrated battery fabricated with SRAL exhibits excellent environmental stability (Movie S1, Supporting Information). It can stably power LED displays even under harsh conditions, such as bending in water (Movie S2, Supporting Information) or violent shaking (Figure 4d,e; Movie S3, Supporting Information), proving the potential of this SRAL integration strategy for practical applications.

## 3 Conclusion 

This work elucidates that the failure mechanism of a directly integrated flexible battery using conventional rigid glue is mainly due to the mismatch between the generated stress and the maximum allowable stress at the adhesive/substrate interface. Accordingly, we have proposed the SRAL strategy, which uses elastic adhesive instead of rigid adhesive to redistribute the maximum stress generated away from the weak adhesive/substrate plane, thereby improving the overall mechanical reliability. The general effectiveness of the SRAL strategy has been confirmed by using the elastic PU with a maximum stretch of 1425% as the elastic adhesive on various flexible substrates. The electrochemical output of the directly integrated flexible battery using SRAL has been demonstrated to be stable under various harsh conditions. The SRAL strategy is expected to be generally effective for other flexible electronics which involves the integration of rigid units on flexible substrates.

## 4 Experimental Section

### Materials

Isophorone diisocyanate (IPDI, 98%), Triethanolamine (TEOA, 97%), and 1,4‐Butanediol (BDO,98%) were supplied by Sigma–Aldrich. Polytetramethylene glycol (PTMG, Mn = 2000 g mol^−1^) was provided by Shanghai Aladdin Biochemical Technology Co., Ltd. (China). The polydimethylsiloxane (PDMS) base and curing agent (Sylgard 184) purchased from Dow Corning were used for the preparation of PDMS films. Ecoflex 00–30 (Smooth‐On, Inc. PA.) was prepared by mixing equal volumes of prepolymers A and B, provided by the supplier. Polystyrene‐block‐poly(ethylene‐ran‐butylene)‐block‐polystyrene (SEBS) was purchased from Sigma–Aldrich. The percentage of polystyrene was 30wt% and the average molecular weight of SEBS was 118 kg mol^−1^. Commercial epoxy adhesive Aradite, and bb‐fox 502 were supplied by Araldite, and Teson T‐99 and Teson 4‐min were supplied by Teson.

### Preparation of Stress‐Redistribution‐Adhesive‐Layer (SRAL)

PTMG2000, TEOA and BDO were dried under vacuum at 110 °C for 6 h before putting them into use. IPDI was kept in a low‐temperature (2−8 °C) freezer until use.

The preparation process of SRAL involved two steps: pre‐polymerization and cross‐linking. Pre‐polymerization stage:PTMG2000, BDO (after vacuum drying), and IPDI with a given ratio (molar ratio 1:1:2.4) were added into a glass bottle with magnetic stirring. The mixture was continuously pumped with nitrogen gas, stirred at 300 r min^−1^, and reacted at 60 °C for 90 min. Crosslinking stage: TEOA (vacuum‐dried) was added to the system after pre‐polymerization with stirring at 200 r min^−1^ for 30 min under a nitrogen atmosphere (molar ratio PTMG2000:BDO:IPDI:TEOA = 1:1:2.4:0.27). After being mixed evenly, the mixture was taken out and dried (80 °C, 24 h).

### Integration of the Cell Arrays

The electrodes were prepared by a conventional slurry coating‐drying process using NCM with the size of 8.0–12.0 µm or LTO with the size of 4.0–7.0 µm as the active materials. The binder was prepared by dissolving PVDF powder (Canrd) into N‐Methyl‐2‐pyrrolidone (NMP, Canrd) to form a 5% solution. For slurry preparation, the active material (NCM or LTO) was mixed with Acetylene black with a mass ratio of 90:5. Then, the calculated volume of 5% PVDF solution was added into the active material/conductor powders to form a slurry containing 5% binder. Slurries were formed by manual grinding and then coated on Al (cathode) or Cu (anode) substrates using four‐sided stainless steel notched rods with a certain gap height. The coated electrodes were dried in an oven at 60 °C for 40 min and then transferred to a vacuum oven and kept at 110 °C for 10 min. In general, the loading thickness of the cathode (excluding Al substrate) was 80.0 ± 8.0 µm. The thickness of the anode (excluding Cu substrate) was 100.0 ± 10.0 µm.

The electrodes were punched with a 12 mm diameter disc punch and weighed with a microbalance with a readability of 0.001 mg. CR2032‐type coin cells were assembled in an argon‐filled glove box using an electrolyte of EC/DMC containing 1.0 mol L^−1^ LiPF_6_.

For forming the cell arrays, the individual cell was interconnected using wires in series or parallel. The silver glue was used to ensure the conductivity and reliability of the contacts.

### Fabrication of Flexible Batteries with SRAL

The flexible PDMS substrate was prepared by mixing the base and curing agent uniformly at a weight ratio of 10:1 followed by aging at 60 °C for 20 min to obtain semi‐cured PDMS. Drop the undried semi‐polymerized PU after chain extension on the desired area on the semi‐cured PDMS substrate. The LR41/CR2032 coin cells were respectively placed onto the semi‐cured PU and slightly tapped to expel trapped gas bubbles. The integrated battery was then kept at 80 °C for 24 h.

### Electrochemical Tests

CV and EIS were tested using a CHI 660E electrochemical workstation. Before the EIS test, the cells were first run for three cycles, charged to the given voltage and then rested for 24 h. The rate and cycling tests were performed on the LANHE CT3001A battery tester. Each individual NCM‐LTO coin cell was charged/discharged in the voltage range of 0–2.7 V. For two cells connected in series, the tested voltage range was 0–4.95 V. For two cells connected in parallel, the voltage range was 0–2.7 V.

### Mechanical Test

To quantify the mechanical property, the flexible substrate of the integrated battery was fixed to the bottom of the stretching machine. The stretching die was used to clamp the adhered coin cell. The coin cell was stretched upward until it was completely detached from the substrate.

### Characterization of the Raw Materials

The phase and morphology of the active materials were characterized by XRD (PANalytical B. V. Empyrean) and SEM (HITACHI SU8010). The composition of the SRAL was analyzed using TG (NETZSCH STA 449F5) and FT‐IR (Thermo Fisher Scientific, Nicolet iS50).

### Finite Element Analysis (FEA)

To analyze the stress distribution of flexible batteries with SRAL, modeling was run using the static structural module in the ANSYS workbench software. For the flexible substrate, the dimension of the flexible substrate was set as 24.0 × 56.0 × 0.3 mm^3^. Two widely used flexible substrates are modeled: PDMS (Young's modulus was set as 2.6692 MPa, Poisson's ratio was set to 0.499) and Ecoflex (Young's modulus was set as 0.43019 MPa, Poisson's ratio was set as 0.48). A cylindrical model with a size of Φ8.9 × 0.5 mm was used for both the SRAL and conventional rigid adhesive layer. The Young's modulus and Poisson's ratio of the SRAL were set as 0.12175 MPa and 0.485. The Young's modulus and Poisson's ratio of the conventional rigid adhesive layer were set as 1000 MPa and 0.39. For each integrated coin cell, it was represented by a cylindrical model with a size of Φ7.9 × 3.6 mm. The battery was made of structural steel with Young's modulus of 20 000 MPa and a Poisson's ratio of 0.3. 3D nonlinear finite element analysis was used to simulate the lateral and vertical deformation of flexible batteries prepared using SRAL, and the stress transfer effect of SRAL, when subjected to deformation, was analyzed, which was realized in the commercial software ANSYS workbench. When simulating lateral deformation, the left side of the substrate was fixed, a 10 mm displacement to the right was applied to the right side of the substrate, and the imprinted surface was applied to record each contact surface (SRAL/Battery, SRAL/substrate, Adhesive Layer /Battery, Adhesive Layer/Substrate) stress distribution. When simulating vertical deformation, fix the substrates on the left and right sides, apply an upward displacement of 10 mm to the battery, and record and analyze the stress distribution of the contact surface.

## Conflict Of Interest

The authors declare no conflict of interest.

## Author Contributions

Y.X. conceived and designed the experimental work and prepared the manuscript. Z.W. carried out the finite element analysis; X.Y. purchased all the chemicals. T.L. helped with SEM characterizations. S.J. helped with XRD tests. T.H. edited the figures. H.J. edited the Supporting Information. X.L. edited the Supporting Movie. W.K. edited the manuscript. Y.H. helped with XRD tests. X.G. supervised the overall project. All authors have given approval to the final version of the manuscript.

## Supporting information

Supporting Information

Supplemental Movie 1

Supplemental Movie 2

Supplemental Movie 3

## Data Availability

The data that support the findings of this study are available from the corresponding author upon reasonable request.
