# Supplementary material for: Elastic Polyurethane as Stress‐Redistribution‐Adhesive‐Layer (SRAL) for Directly Integrated High‐Energy‐Density Flexible Batteries
Source: Adv Sci (Weinh). 2024 Jun 3;11(29):2401635. doi: 10.1002/advs.202401635 (PMC11304273; doi:10.1002/advs.202401635)
Supplement: Supplementary file 1 — Supporting Information [file ADVS-11-2401635-s004.docx]

Supporting Information

Elastic polyurethane as stress-redistribution-adhesive-layer (SRAL) for directly integrated high-energy-density flexible batteries

*Yige Xiong, Zhongjie Wang, Xiaohui Yan, Taibai Li, Siqi Jing, Tao Hu, Huixin Jin, Xuncheng Liu, Weibo Kong, Yonglin Huo, Xiang Ge**

Y. G. Xiong, Z. J. Wang, X. H. Yan, T. B. Li, S. Q. Jing, T. Hu, Prof. H. X. Jin, Prof. X. C. Liu, Y. L. Huo, Prof. X. Ge

Department of Materials and Metallurgy, Guizhou University, Guiyang, Guizhou 550025, China,

E-mail: xge@gzu.edu.cn

Dr W. B. Kong

College of Polymer Science and Engineering, Sichuan University, Chengdu, 610065, China

E-mail: kwb@scu.edu.cn

**Table of Contents**

**Figure S1**: Schematic diagram of the rigid integration method.

**Figure S2:** Stress distribution during vertical deformation of a flexible substrate is shown.

**Figure S3**: FTIR spectra of the PTMG, IPDI, BDO, TEOA and PU.

**Figure S4**: TG-DTG curve of PU.

**Figure S5**: The stress-strain curves of PDMS (polydimethylsiloxane), Ecoflex, and PU (polyurethane).

**Figure S6:** XRD patterns of the Li_3_MnCoNiO_6_ (NCM) and the Li_4_Ti_5_O_12_ (LTO).

**Figure S7:**. SEM images of the NCM and the LTO.

**Figure S8**: EIS spectrum of the materials tested in half-cells and full-cells.

**Figure S9**: CV curves of the materials tested in half-cells and full-cells.

**Figure S10**: Rate capability of the materials tested in half-cells and full-cells.

**Figure S11**: Cycling performance and charge/discharge curves of the materials tested in half-cells and full-cells.

**Supporting Figures**

**
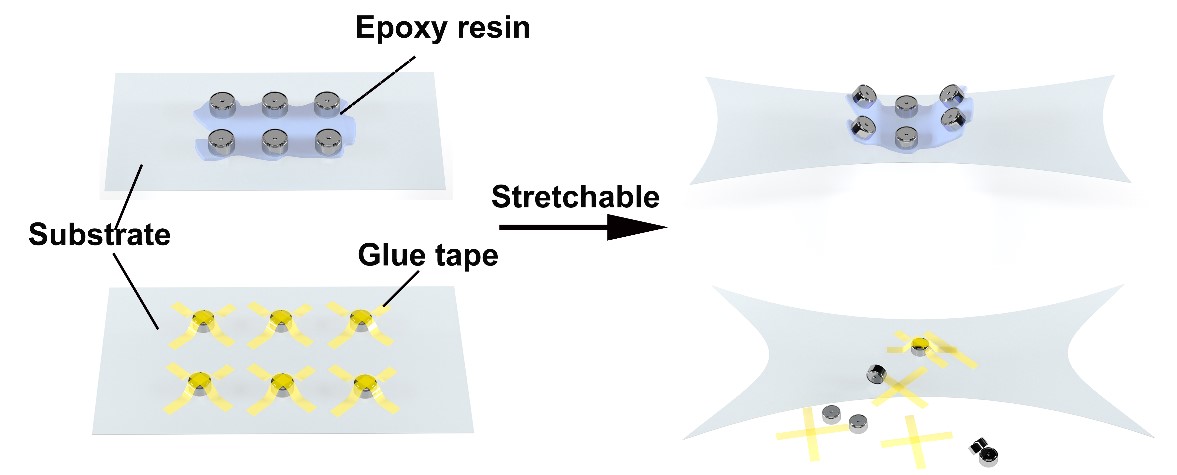
**

**Figure S1.** Schematic illustration showing that conventional integration methods using rigid adhesive can not provide robust integration.


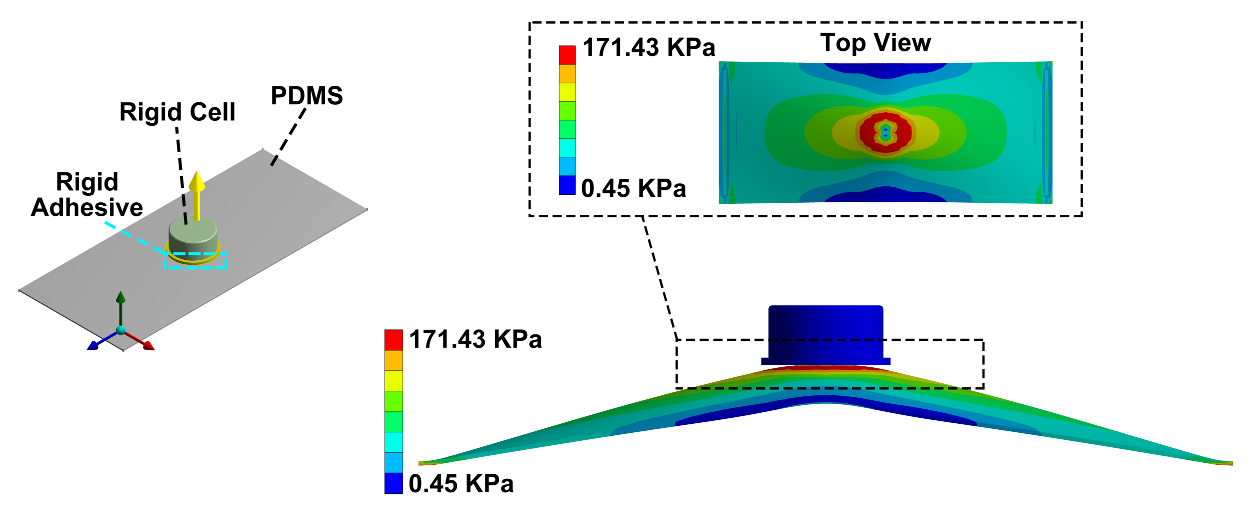


**Figure S2.** Stress distribution during vertical deformation of a rigid cell integrated on flexible substrate using the conventional rigid adhesive layer.


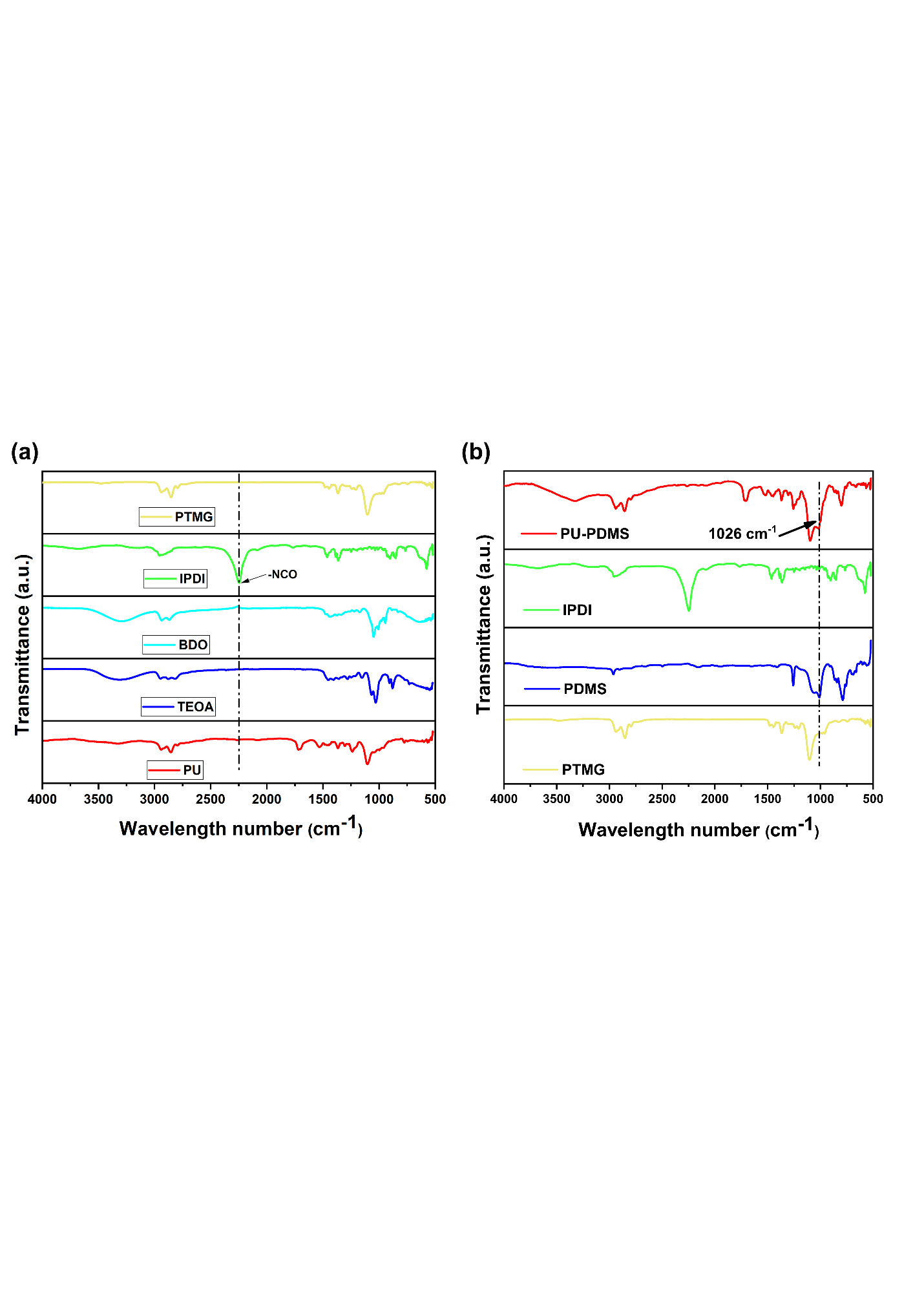


**Figure S3.** a) FTIR spectra of the PTMG, IPDI, BDO, TEOA and PU. b) FTIR spectrum of the PU-PDMS.

As shown in Figure S3a, for the infrared spectrum of isophorone diisocyanate (IPDI), there is a prominent peak at 2270 cm^-1^, which is attributed to the vibrational absorption of the isocyanate group (-NCO). However,this peak is absent for PU. This indicates that the isocyanate groups (-NCO) have been completely comsupted during the synthesis of PU.^[1]^ At the same time, the absorption peak appears in the range of 1500-1750 cm^-1^, corresponding to the stretching vibration of C=O bond and the deformation vibration of N-H bond, which further confirms the formation of carbamate in PU. To elucidate the adhesive mechanism between PU and PDMS, we have prepared a sample (PU-PDMS) by directly introducing PDMS into the mixture of PTMG and IPDI during the preparation of PU. The FTIR spectrum of the PU-PDMS is given in Figure S3b. Compared to the original PU and PDMS materials, PU-PDMS did not exhibit any additional signals. The prominent peaks observed in PU-PDMS were also present in PDMS and PU individually. Therefore, we believe that no new chemical bonds were formed between PDMS and PU. The interaction between PU and PDMS involved the entanglement of cross-linked molecules, which is also the reason for the significant enhancement of adhesion between PDMS and PU.


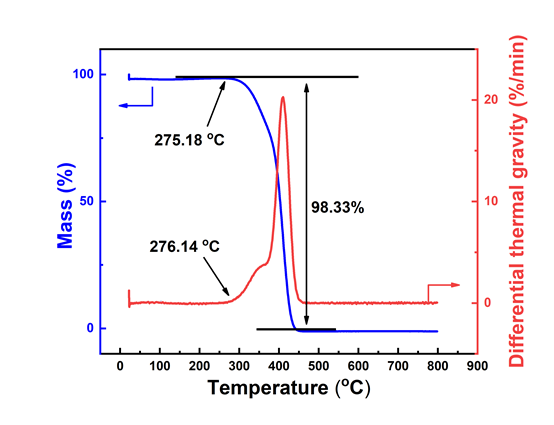


**Figure S4.** TG-DTG curve of PU.


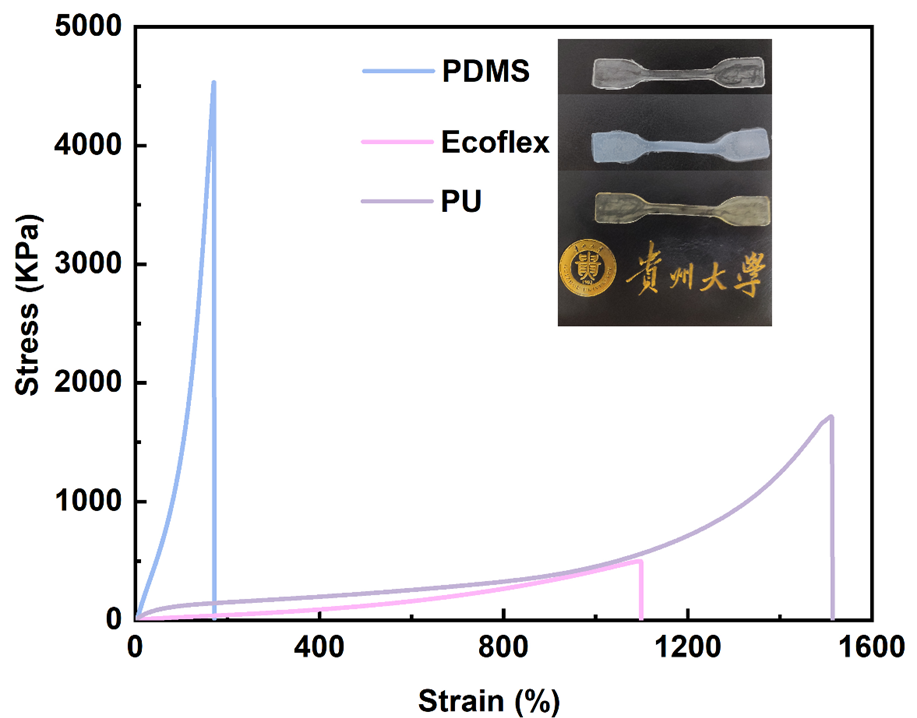


**Figure S5.** The stress-strain curves of PDMS, Ecoflex and PU. The inset shows the digital picture for the sample used for tensile stretching.

**
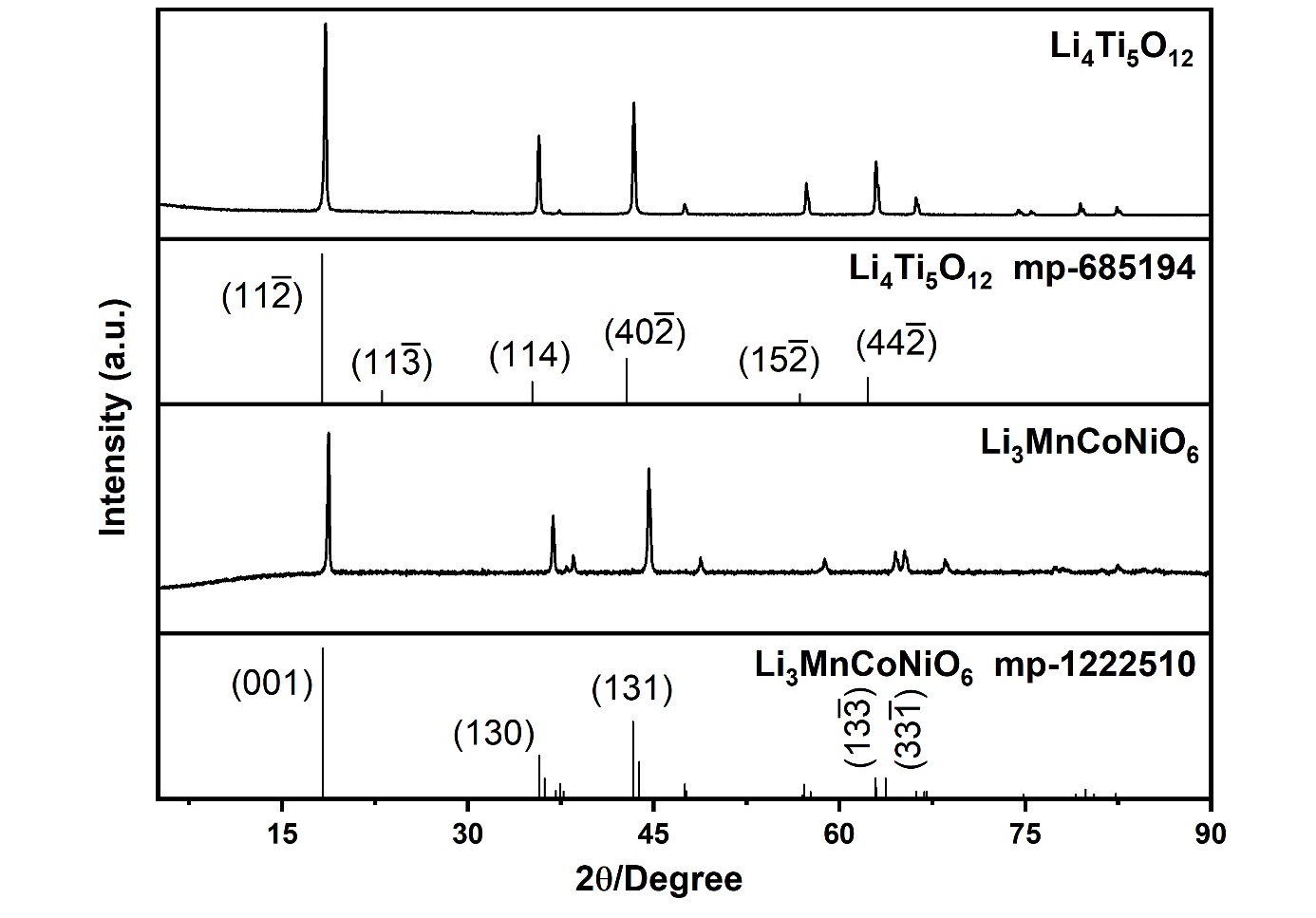
**

**Figure S6.** XRD patterns of the Li_3_MnCoNiO_6_ (NCM) and the Li_4_Ti_5_O_12_ (LTO).


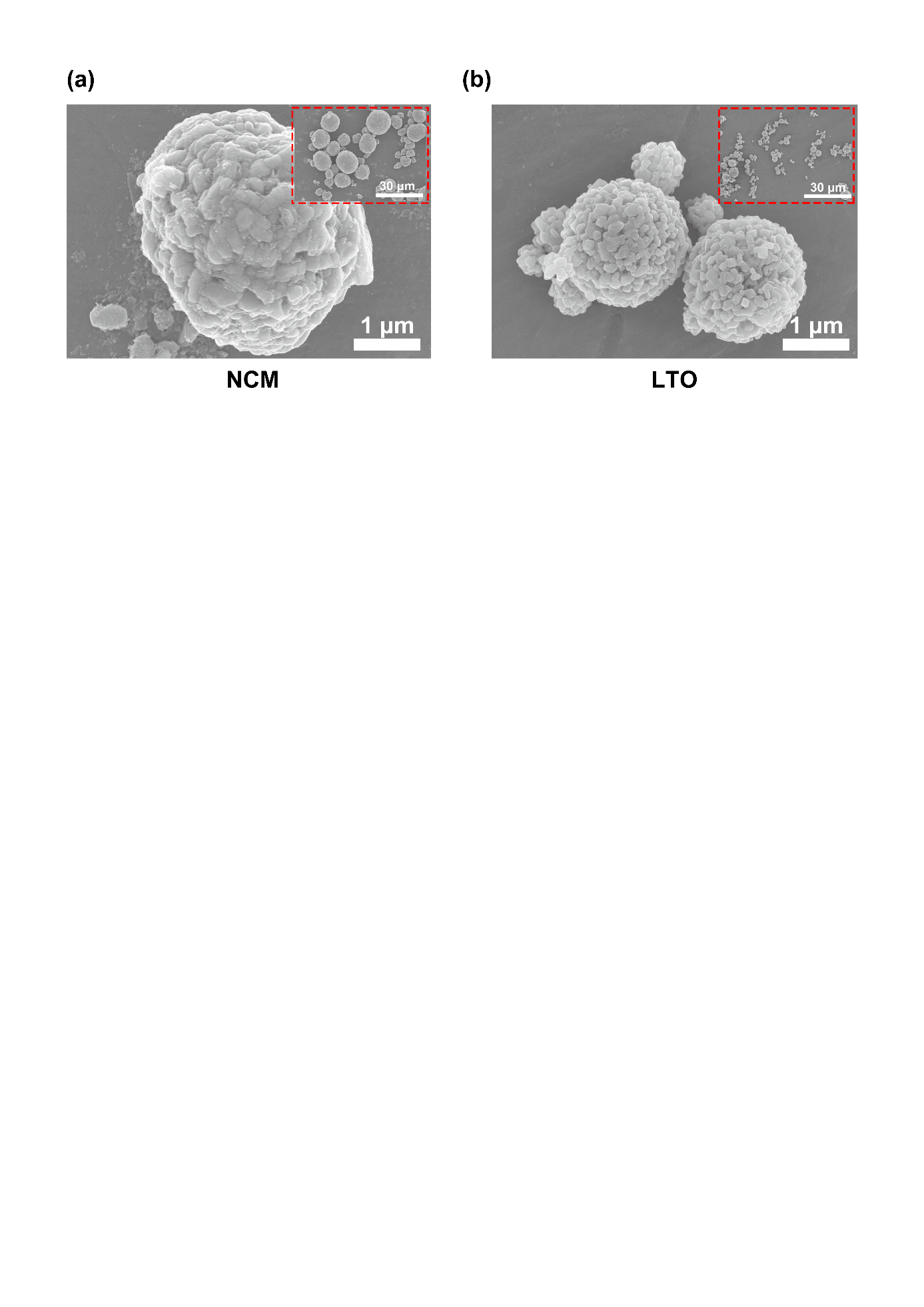


Figure S7. SEM images of the NCM and the LTO.


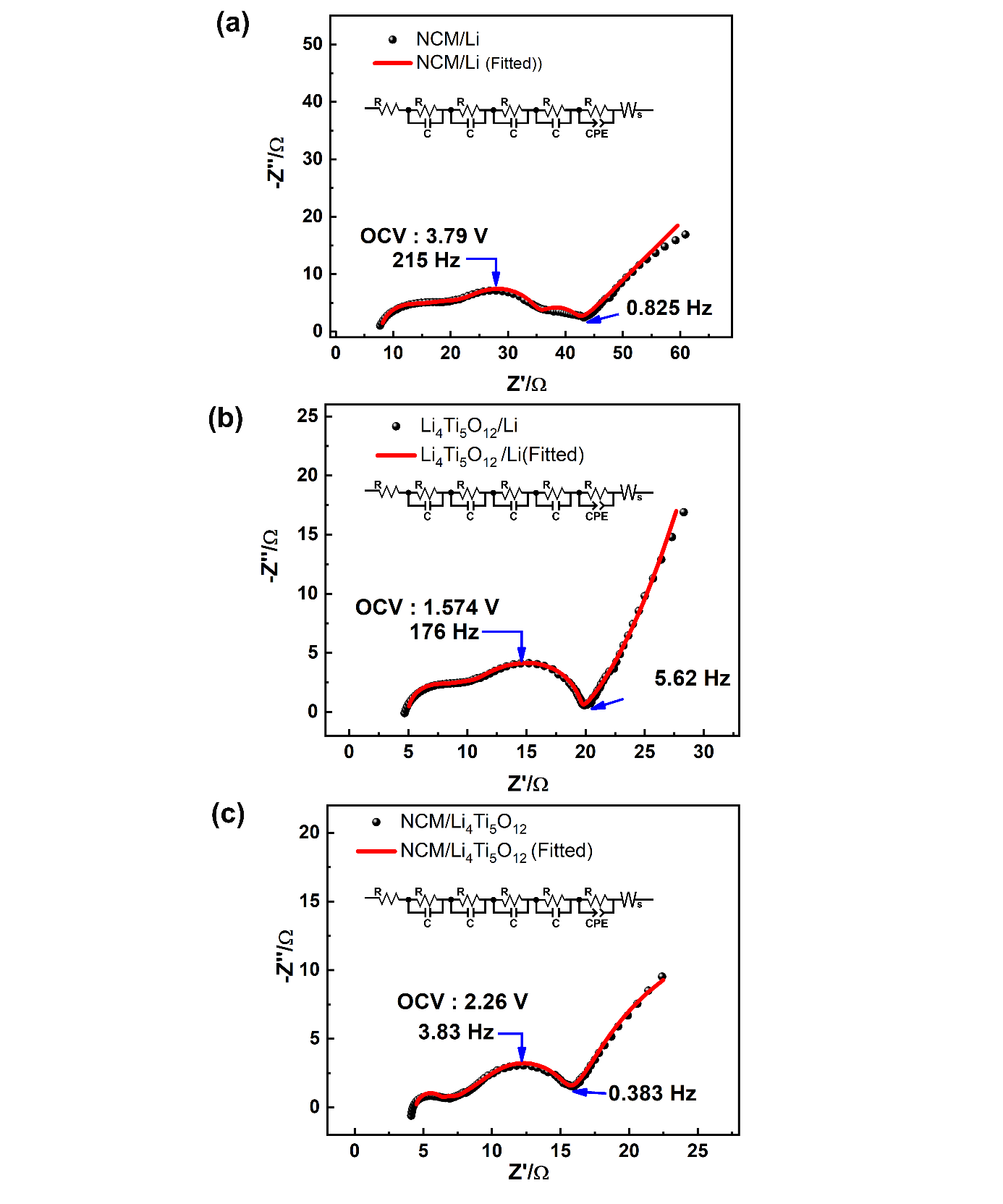


**Figure S8.** **EIS spectrum of the materials tested in half-cells and full-cells.** EIS measurement for NCM/Li (a), LTO/Li (b) and NCM/LTO (c).

To gain a better understanding of the electrochemical kinetics for the electrodes, we conducted electrochemical impedance spectroscopy (EIS) tests on the CHI 660E electrochemical workstation. Prior to the EIS measurements, the battery underwent three charge/discharge cycles and was then left to stabilize until the open-circuit potential reached a steady state.

The Nyquist plots were obtained by applying a perturbation voltage of 5 mV within the frequency range of 0.1 Hz to 100 kHz. All Nyquist plots exhibited a high-frequency semicircle, a mid-frequency semicircle, and a long low-frequency line. The semicircle in the high-mid frequency region represents the charge transfer impedance of the electrode associated with the Faradaic reaction.^[2]^ The inclined line in the low-frequency region represented the Warburg impedance, indicating the diffusion capability of lithium ions in the inactive materials.^[3]^ The Levi's equivalent circuit simulator based on Voigt-type modeling, the generalized Frumkin and Melik-Gekazyan impedance combination is used for distinguishing the impedance components in the obtained EIS data.^[4]^ The equivalent circuit is illustrated in Figure S8. Moreover, considering the differences in material porosity, we employed constant phase elements (CPE) instead of pure capacitors at the parallel connection of the charge transfer resistance (Rct).^[5]^ Additionally, we removed the C_int_ component from the Levi model, which represents the capacitance of graphite electrode insertion, as it was not applicable to the present study due to the different lithium-ion storage mechanisms for LTO. The fitted data based on the modified equivalent circuit (lines in Figure S8) were found to be in good agreement with the measured impedance data (dots in Figure S8). The results indicate that both LTO and NCM electrodes possess favorable charge and ion conductivity, as evidenced by their lower charge transfer resistance and lithium-ion resistance through the SEI layer. The NMC/LTO full cell exhibits excellent electrochemical kinetics, making it a preferred choice for geometrically flexible batteries.


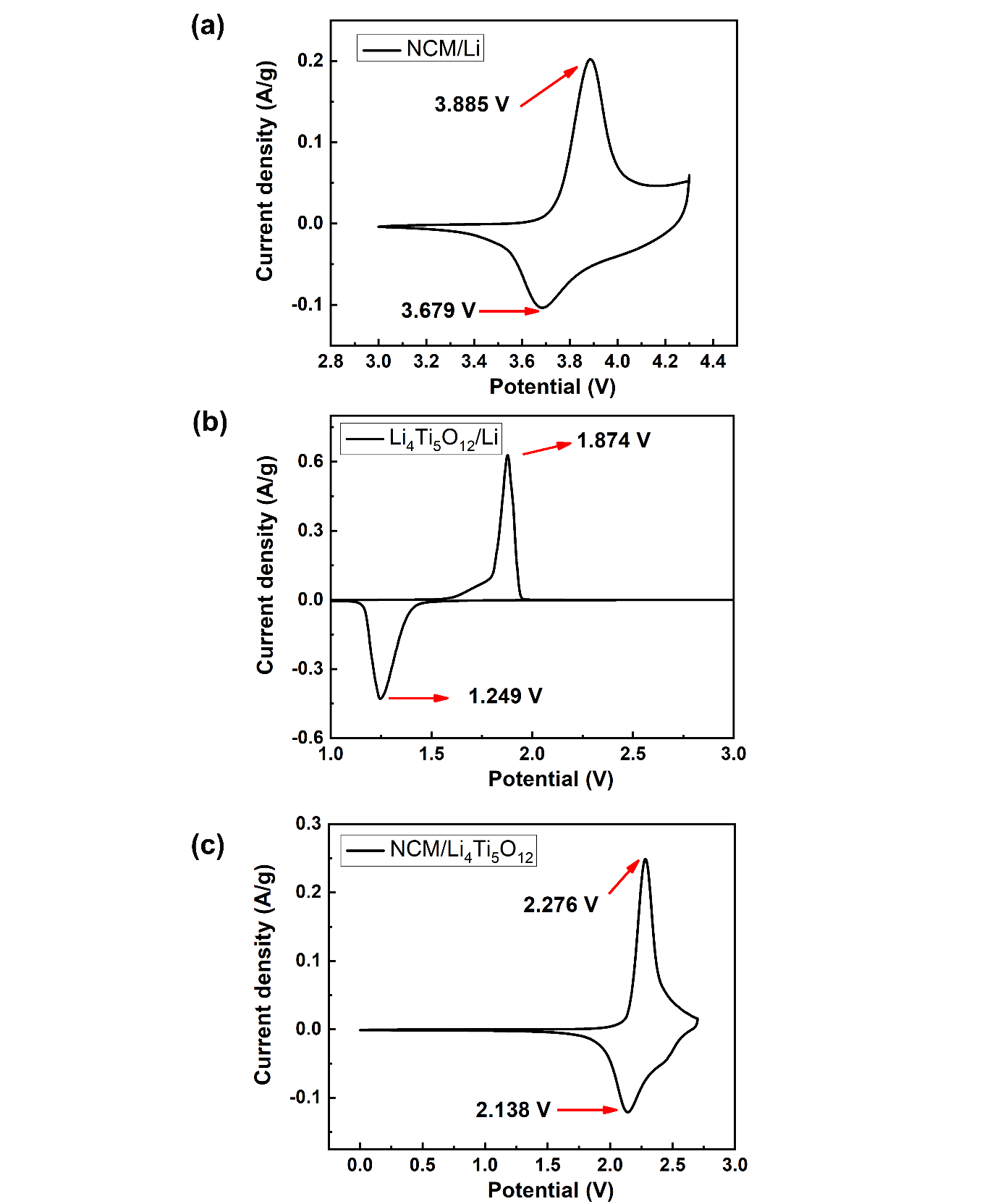


**Figure S9.** CV curves of NCM/Li (a), LTO/Li (b) and NCM/LTO (c), where the loading of NCM/LTO is based on NCM side.


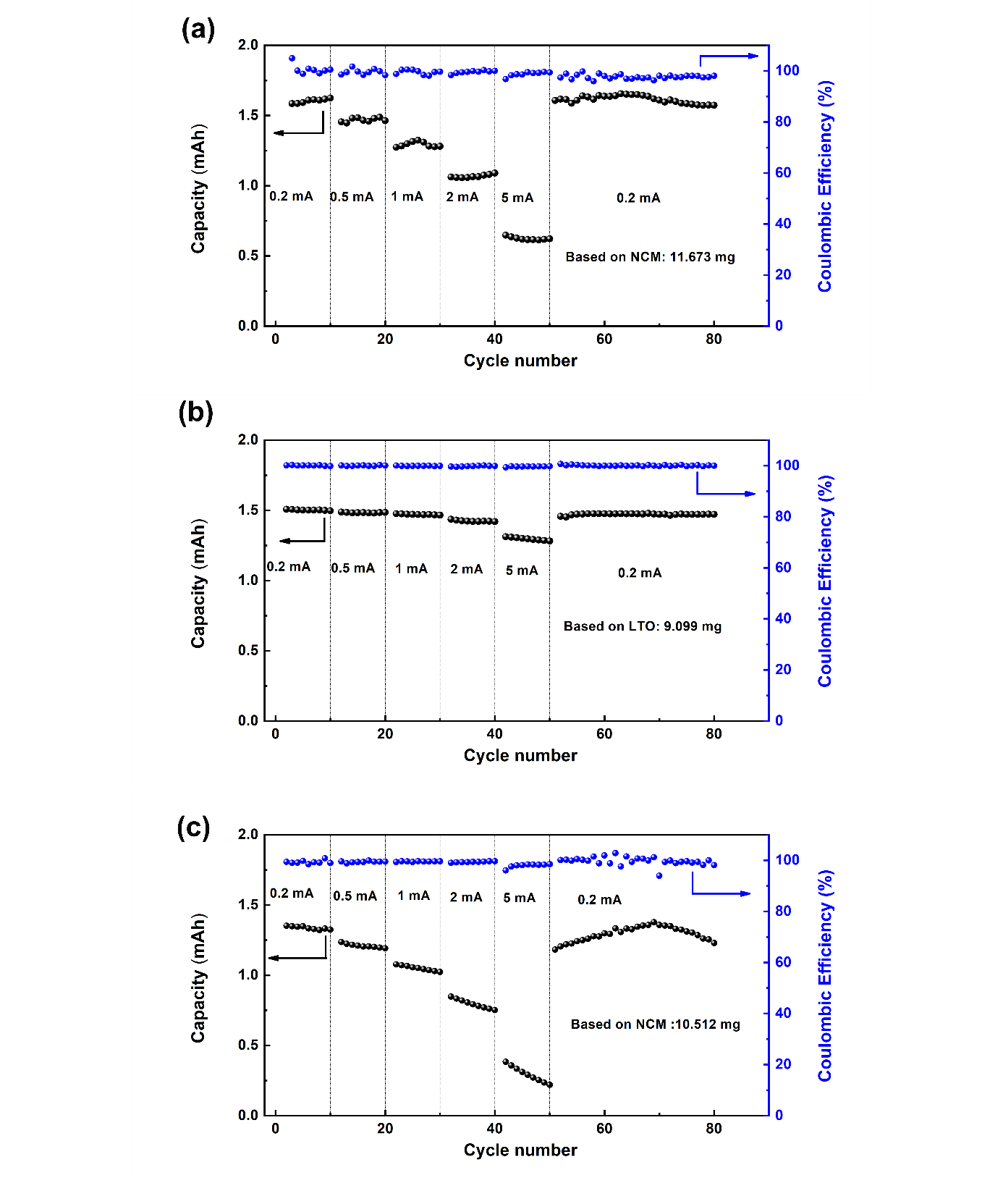


**Figure S10.** Rate capability of NCM/Li (a), LTO/Li (b) and NCM/LTO (c).


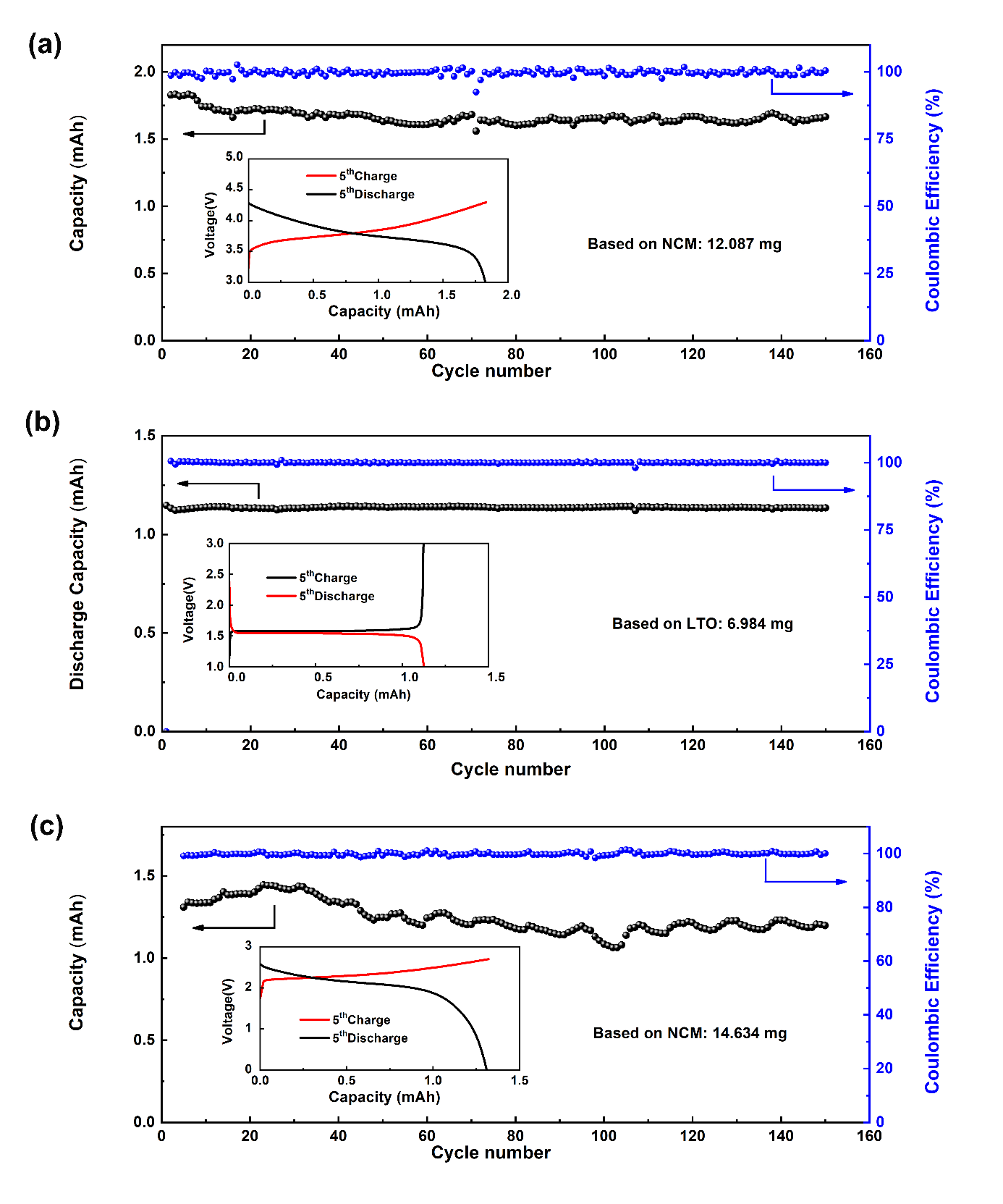


**Figure S11.** Cycling performance and charge/discharge curves of NCM/Li (a), LTO/Li (b) and NCM/LTO (c).

[1] M. Sadeghi, M. A. Semsarzadeh, M. Barikani, B. Ghalei, *J. Membr. Sci.* **2011**, 385-386, 76.

[2] Y. Lei, D. Han, J. Dong, L. Qin, X. Li, D. Zhai, B. Li, Y. Wu, F. Kang, *Energy Stor. Mater.* **2020**, 24, 319.

[3] D. Liu, X. Fan, Z. Li, T. Liu, M. Sun, C. Qian, M. Ling, Y. Liu, C. Liang, *Nano Energy* **2019**, 58, 786.

[4] a) M. Levi, D. Aurbach, *J. Phys. Chem. B* **1997**, 101, 4630; b) D. Aurbach, I. Weissman, A. Schechter, H. Cohen, *Langmuir* **1996**, 12, 3991.

[5] X. Ge, C. D. Gu, X. L. Wang, J. P. Tu, *J. Phys. Chem. C* **2014**, 118, 911.
